# Systematic optimization of culture media for maintenance of human induced pluripotent stem cells using the response surface methodology

**DOI:** 10.1016/j.heliyon.2024.e32558

**Published:** 2024-06-09

**Authors:** Seyedmilad Mousavi Mirkalaei, Shirin Farivar

**Affiliations:** Department of Cell and Molecular Biology, Faculty of Life Sciences and Biotechnology, Shahid Beheshti University, Tehran, Iran

**Keywords:** *Human induced pluripotent stem cells*, *response surface methodology*, *Fibroblast growth factor*, *Cell density*

## Abstract

The application of human induced pluripotent stem cells (hiPSCs) provides tremendous opportunities in cell therapy. However, culturing these cells faces many practical challenges, including costs associated with cell culture media and the optimization of cell culture conditions. Providing an optimized culture platform for hiPSCs to maintain pluripotency and self-renewal and generate cost-effective and robust therapeutics is an immediate requirement. This study used the design of experiments and the response surface methodology, a powerful statistical tool, to generate empirical models for predicting the optimal culture conditions of the hiPSCs. Pluripotency and cell proliferation were applied as read-outs to determine the optimal concentration of basic fibroblast growth factor (bFGF) and cell density. One model was defined to predict pluripotency and cell proliferation in terms of the predictor variables of the bFGF concentration and cell seeding density. Predicted culture conditions to maximize maintaining cell pluripotency were successfully validated. The present study's findings provide a novel approach that can potentially allow controllable hiPSC culture routine in translational research.

## Highlights

• Application of response surface methodology optimized bFGF and cell density for hiPSC culture.
• The RSM approach minimizes experiments, offering a cost-effective and controllable hiPSC culture.
• This Study identified optimal conditions (bFGF 111 ng/ml, 70,000 cells/cm^2^) for hiPSC expansion.
• Predicted conditions (bFGF 130 ng/ml, 70,000 cells/cm^2^) maintained pluripotency.
• Optimal conditions enhance pluripotency marker expression, critical for translational research.

## Abbreviations

bFGF: Basic fibroblast growth factor
CCD: Central composite design
DMSO: Dimethyl sulfoxide
DoE: Design of experiments
hESCs: Human embryonic stem cells
hiPSCs: Human induced pluripotent stem cells
MTT: 3-[4,5-dimethylthiazol-2-yl]-2,5 diphenyl tetrazolium bromide
NCR: Nuclear-cytoplasmic ratio
OD: Optical density
qRT-PCR: Quantitative reverse transcription PCR
RSM: Response surface method

## 1 Introduction

Human induced pluripotent stem cells (hiPSCs) are one of the valuable recent discoveries that can open up unprecedented opportunities in different aspects of clinical therapy such as regenerative medicine [1]. Reprograming adult somatic cells into the hiPSCs can overcome immunological barriers and ethical related to the use of embryonic stem cells (ESCs) [2,3]. Although the hiPSCs can play numerous medical roles, a large number of them with high quality are needed during most of these applications [4,5]. According to the most current cell therapy protocols and those under development, at least 10^8^–10^10^ cells are required per patient [6]. Therefore, the efficient expansion of these cells on a large scale under well-defined culture conditions and safety-reliable is one of the necessities that has been the focus of recent research [7]. During expansion, cells undergo growth through division, making it crucial to maintain their quality and condition to avoid disrupting homologous self-renewal [8]. Consequently, when expanding stem cells, specific optimized media and conditions are employed. The optimal culture conditions for hiPSCs cultivation still rely on empirically formulated media, despite rapid advances in the hiPSCs technology [9]. The empirically formulated culture media could successfully maintain the survival and expansion of hiPSCs [10,11]. However, the earlier hiPSC culture systems have been faced with several challenges. For instance, the presence of xenogeneic components in culture systems increases the risk of pathogenic and immune rejection [12,13]. The major obstacle in stem cell culture is their limited survival time [14,15]. To address this, there is a need for a growth factor that can promote sustained growth and proliferation [15]. The basic fibroblast growth factor (bFGF) is a crucial growth factor known for its ability to support the growth and proliferation of cells, including stem cells [16]. Previous studies have shown that hiPSCs rely on exogenous bFGF for both self-renewal and differentiation into various somatic cell types [17].

The seeding density of stem cells significantly impacts pluripotency cultivation. Epigenetic memory, pluripotency, and differentiation potential are all influenced by the initial density at which stem cells are seeded [18,19]. Optimizing the seeding density can enhance both the yield and purity of stem cells.

These components also can cause limited insight into the understanding of the underlying molecular mechanisms, which hinders the clinical application of hiPSCs. In addition, lack of affordability and compatibility among the categories are among other defects of these cultivation systems [20]. It suggests that well-designed optimization steps and evaluating the interactions between manifold components can solve problems associated with empirically formulated media [9].

One of the best mathematical techniques used to determine the optimal set of conditions across many different changeable parameters is the statistical design of experiments (DoE) [21]. The main advantage of the DoE approach is the capacity to reduce the number of experiments required to identify an optimal set of conditions. The DoE strategy can decrease the time and costs of the optimization of numerous bioprocessing procedure variables and provide much information about the process with fewer experiments [22,23]. The DoE has been applied in various fields such as microbiology [24], drug optimization [25], and engineering [10]. However, applying DoE to optimize hiPSCs culture conditions is at the beginning of the research [9]. To the best of our knowledge, the combined use of cell density and bFGF as effective factors for optimizing culture conditions and regulating pluripotency has not been explored. This study aimed to optimize the levels of bFGF and cell density in the culture medium by using the response surface method for improving hiPSCs cultivation conditions.

## 2 Materials and methods

### 2.1 The RSM design of experiments

**Fig. 1:**
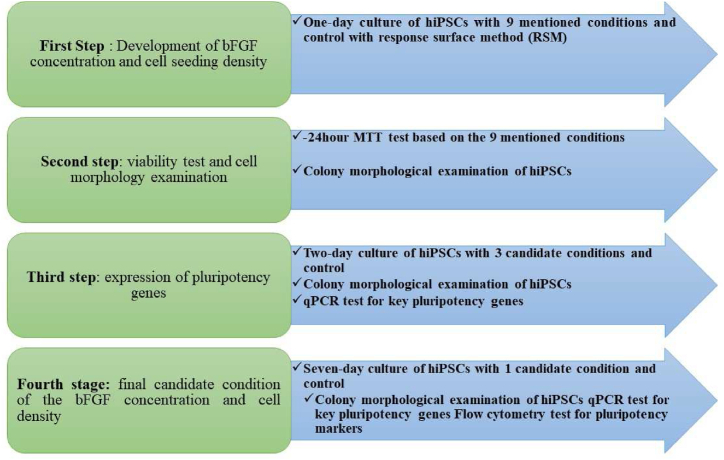
Diagram of the experiment stages in this study.

The three-surface, two-factor central composite design (CCD) was used to evaluate the effects of two factors, the bFGF concentration and seeding density [26] (Fig. 1). To each factor, three levels were assigned which were coded as low (−1), medium (0), and high (+1). For estimation of all possible interactions, a full factorial design, comprising a set of 9 different conditions was generated in Design-Expert v10 DoE software (Stat-Ease, Inc., Minneapolis, MN) at least in 3 independent experiments (Supplementary Data 1). The MTT test and colony morphological examination were conducted on hiPSCs in all nine experimental conditions and in the control group (50,000 cells/cm^2^ without bFGF treatment^)^ after 24 h to determine the optimal cell proliferation and viability conditions. In the research model, a boundary was set up to optimize the survival and proliferation of cells. This boundary was determined based on the results of the MTT test, which measures the metabolic activity of cells and is often represented as optical density (OD). According to the results of the MTT test, the culture of the hiPSCs was performed in 3 selected conditions and a control group (70,000 cells/cm^2^ without bFGF treatment) for 48 h. Pluripotency gene expression and colony morphology of the hiPSCs were examined during this time. Subsequently, hiPSCs were cultured for seven days in one selected condition (the one with the proliferation and viability response closest to the predicted optimum point) and a control group (70,000 cells/cm^2^ without bFGF treatment). Key pluripotency gene expression, flow cytometric analysis of pluripotency markers, cell apoptosis, and colony morphology of the hiPSCs were evaluated.

### 2.2 The hiPSCs culture and maintenance

In this study, we used the hiPSC line UMN PCBC16iPS, derived from neonatal dermal fibroblasts (UMNi001-A: CVCL_A4EW) [27], obtained from the Basic Medical Sciences Research Center, Histogenotech Company, Tehran, Iran. Gene expression analysis for Oct4, Sox2, and c-Myc was performed 24 h after cell culture for authentication. The cells were tested for mycoplasma contamination by PCR and were confirmed negative within six months. In this study, a single layer of mouse embryonic fibroblasts (MEFs) treated with mitomycin C (Merck, Germany) was used as a feeder layer to culture cells in a special culture plate. This approach provided extracellular matrix constituents and a nurturing environment that facilitated the proliferation and maintenance of cells. The medium was changed every three days, and cells were passaged using EDTA (Bualisina, Iran) every week. The hiPSCs were washed with 1 ml of PBS. Then, 1 mL of 0.5 mM EDTA per well was added and incubated for 3–5 min at room temperature [28]. The media including DMEM F12 culture medium with 20 % KOSR (Sigma–Aldrich, UK), 2 mM glutamine, 1 % NEAA, and 0.1 mM beta-mercaptoethanol (Sigma–Aldrich, UK) was added. After partial dissociation of the cells by up-and-down pipetting, the hiPSCs were plated on a new 6-well plate and maintained at 37 °C with 5.2 % CO2 and 90 % humidity [29].

### 2.3 Determination of cell density

The colony density of cells was estimated based on the size of each colony (the number of cells in the colony based on the dimensions of a cell, which is about 20 μm) using ImageJ software [30,31]. The cell density of hiPSCs is estimated based on colony formation rather than traditional cell counting, given the presence of feeder cells. In this way, after separating the hiPSCs colonies from the feeder cells, the formula for calculating the number of cells in each hiPSC colony based on the sphere volume formula and desired cell density can be expressed as the following:

$$\text{Number}\;\text{of}\;\text{cells}=\left(\text{Desired}\;\text{cell}\;\text{density}\;\text{per}\;{\text{cm}}^{2}\right)\;*\;\left(\text{Area}\;\text{of}\;\text{culture}\;\text{vessel}\right)\;/\;\left(\text{Number}\;\text{of}\;\text{cells}\;\text{per}\;\text{colony}\right)$$

where, the desired cell density per cm^2^ represents the desired number of cells per unit area, which can be 30,000, 50,000, or 70,000 cells per cm2. The area of the culture vessel represents the total area available for cell culture, which in this case is 9.6 cm^2^ for a 6-well plate culture. The number of cells per colony is the estimated number of cells in each hiPSC colony, which is calculated based on the sphere volume formula considering the colonies as spheres with dimensions of 100 μm in diameter, and estimated to contain about 500 cells.

#### 2.3.1 MTT assay

The 3-(4,5-Dimethylthiazol-2-yl)-2,5-diphenyltetrazolium bromide (MTT) assay was used to evaluate the viability of the hiPSCs in different conditions in comparison with the control [32]. In brief, the hiPSCs in all conditions were exposed to MTT solution (5 mg/mL in DMEM)(Alfa Aesar, USA) and then kept at 37 °C for 3.5 h for conversion of MTT to formazan crystals. After that, the medium was removed, formazan crystals were dissolved by 100 μL DMSO for 15 min at 37 °C with 5.2 % CO2 and 90 % humidity, and the optical density was measured at 570 nm by a Bio-Tek Instruments, Winooski, VT). The OD values were compared between the control and experimental groups, in which each group underwent three repetitions of the experiment.

### 2.4 Cell and colony morphometry

The Eclipse TE2000-S inverted microscope (Nikon), which is equipped with E-Plan 10 × and 20 × objectives, a long working distance, and a super high-pressure mercury lamp, was utilized for live cell and cell colony imaging. Photographic images were captured through a Nikon DXN1200F digital camera controlled by the EclipseNet software (version 1.20.0 build 61). Morphometric parameters, including cell and nucleus area, nuclear-cytoplasmic ratio (NCR), and colony size were computed using ImageJ software (version 1.45) [33,34]. Positive control was established by subjecting a cell group to a differentiation-inducing cytokine cocktail.

### 2.5 Quantitative real-time PCR

Cultures of hiPSCs in different conditions in 6-well plates were washed twice in PBS. Then, the cells were exposed to 1 ml of Trizol for 10 min. Trizol-extracted RNA (Sinaclon, Iran)) was then transferred to a 1.5 ml microtube and their quality was assessed by NanoDrop1000. The extracted RNA was stored at −80 °C. The extracted RNA was reverse transcribed by a High-Capacity cDNA Reverse Transcription Kit (Applied BiosystemsTM, Yokohama, Japan). Real-time quantitative reverse transcription PCR (qRT-PCR) was done by the StepOnePlus system (Thermo Fisher Scientific, US). To measure the expression of specific pluripotency genes, Biosystems' TaqMan assay was applied that includes OCT4 (assay no.:At02611156_m1), NANOG (assay no.: HS02387400_g1), SOX2 (assay no.:Hs04234836_s1), and GAPDH (assay no.: Hs03929097_g1). Normalization of all quantitative gene expression data was done to the expression levels of GAPDH. The relative fold changes (2^-△△Ct^) in the transcript expression of the cells were calculated [35].

### 2.6 Flow cytometric analysis of intracellular and surface pluripotency markers

Immunostaining of intracellular (Sox2) and surface (SSEA4) pluripotency markers was performed as described previously [36]. Briefly, the hiPSCs were twice washed with PBS and fixed in paraformaldehyde (4 % in Hanks' balanced saline solution [HBSS]; Gibco, Grand Island) for 10 min at room temperature. Then, the cells were washed in HBSS and incubated with primary antibodies Sox2 (IC2018A; R&D Systems Inc., Minneapolis, MN) and 1:400; SSEA4 (catalog no. 4755; Cell Signaling Technology). After that, the cells were washed three times with PBS and assessed by immunopositive cell markers using a FACS Canto II flow cytometer (BD Biosciences, Franklin Lakes, NJ, USA). The data obtained were analyzed using FlowJo software (v10, Tree Star).

### 2.7 Cell apoptosis

The hiPSCs apoptosis was quantified using an AnnexinV-FITC/PI apoptosis kit (BioLegend, San Diego, CA) based on the supplier's protocol. Briefly, the cells were detached by using EDTA and collected and rinsed with cell staining buffer (BioLegend). The cells were resuspended in 100 μL Annexin-binding buffer, and then 2.5 μL Annexin V-FITC was added. After 15 min incubation at room temperature, 2.5 μL propidium iodide (PI) was added to the cell suspension and incubated for 10 min in the dark at room temperature. The FITC or PI-positive cells were determined by flow cytometer (BD Biosciences, Franklin Lakes, NJ, USA) by appropriate filters [37].

### 2.8 Statistical analysis

The normality of data was tested by the Shapiro-Wilk test. The differences were analyzed by independent *t*-test or one-way ANOVA followed by Tukey's multiple comparisons post hoc tests. Kruskal Wallis test was used to analyze non-parametric data. The difference was considered statistically significant at p < 0.05 and all quantitative data were presented as mean ± standard deviation.

## 3 Results

### 3.1 First step: development of bFGF concentration and cell seeding density

Based on the response surface method (RSM), nine conditions were generated in Table 1. The examination of the independent variables indicates that a quadratic model (refer to Table 2 and Fig. 1) can accurately describe the optical density (OD) of cells. In this study, the first variable (A) is bFGF and the second variable (B) is seeding density. Regression analysis was performed to fit the response function of proliferation and viability of hiPSCs (measured by OD). The final empirical models in terms of coded factors, after excluding the insignificant terms for bFGF concentration (A) and seeding density (B), are as follows:

hiPSCssurvivalrate=393.56+117.17×A+186.00×B+51.00AB+95.67B2

**Table 1:** Central composite design for optimizing culture media to maintain the survival rate of hiPSCs.

Run	Factor 1 A:bFGF	Factor 2 B: Cell seeding Density (cells/cm^2^)	Response OD (570 nm)
**1**	8	30	247
**2**	92	50	513
**3**	92	30	369
**4**	50	50	408
**5**	8	50	258
**6**	50	70	672
**7**	50	30	292
**8**	8	70	513
**9**	92	70	839

**Table 2:** The variance analysis of main responses of hiPSCs survival rate.

Model	3.187E+05	5	63730.76	228.21	0.0005	significant		
A-bFGF	82368.17	1	82368.17	294.95	0.0004			
B-Seeding Density	2.076E+05	1	2.076E+05	743.31	0.0001			
AB			10404.00	1	10404.00	37.26	0.0088	
A^2^			1.39	1	1.39	0.0050	0.9482	
B^2^			18304.22	1	18304.22	65.55	0.0039	
Residual			837.78	3	279.26			
Cor Total			3.195E+05	8				

**Fig. 2:**
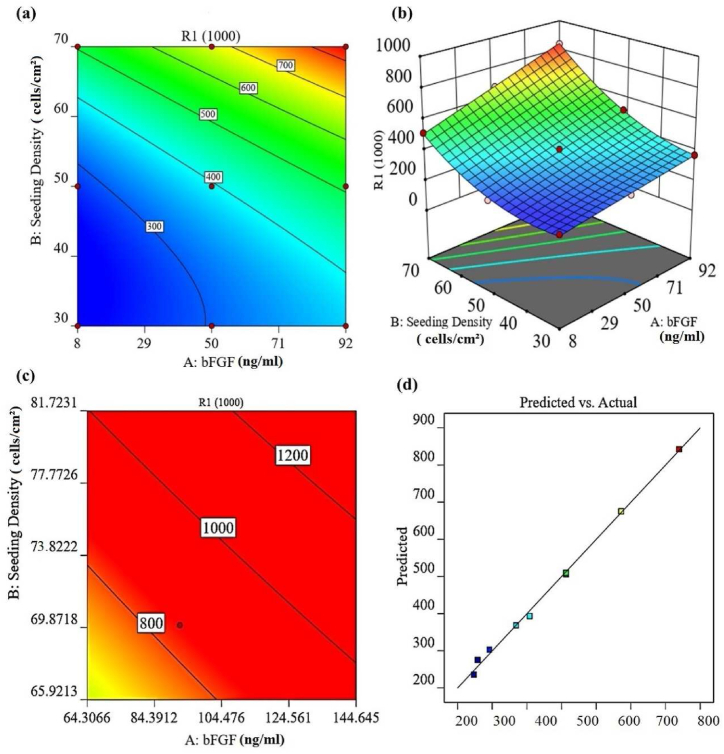
A contour plot (a) and its corresponding response surface plot (b) show the interaction between bFGF concentration and cell seeding density. These were obtained by measuring cell proliferation and survival using the MTT test. The optimum point of interaction between bFGF concentration and cell seeding density is shown in (c). (d) Scatter plot that shows the predicted versus actual values for cell viability based on optical density (570 nm). R1: Response (OD).

The response surface analysis and software enabled the identification of optimal conditions for cell proliferation. Specifically, a bFGF concentration of 50 ng/ml and a cell density of 50,000 were found to be the starting point for achieving maximum proliferation as confirmed by MTT testing (Fig. 2 a-b) (see Supplementary Data 2). The predictive outcomes of the model indicate that the optimal conditions for stem cell culture entail a bFGF concentration of 130 ng/ml and a cell density of 70,000 cells/cm^2^ ((Fig. 2 c-d). Conversely, according to the MTT test results, the highest survival rate occurred at a concentration of 92 ng/ml and a density of 70,000 cells/cm^2^. Aligned with the robust proliferation exhibited by stem cells, the model anticipates heightened cell proliferation with increased cell density and bFGF concentration. Nevertheless, scrutiny of cell morphology at bFGF concentrations surpassing 130 ng/ml and cell densities exceeding 70,000 cells/cm^2^ reveals distinct indications of differentiation, proliferation, and apoptosis (Table 3). A threshold limit was established after reaching densities of 70,000 cells/cm^2^ in which cells were differentiated. Consequently, the experimental design for the second stage incorporated bFGF concentrations of 92, 111, and 130 ng/ml as the minimum, intermediate, and maximum concentrations, respectively.

**Table 3:** Morphometric Indices of iPSCs and Colonies Versus Differentiated Cells Under Varying Experimental Conditions (n = 40) (Mean ± minus the standard error),Statistical significance was assessed using a Mann-Whitney *U* test, with p-values less than 0.05 denoted by an asterisk (*), indicating a significant difference compared to differentiated cells.

Cell condition	Nucleus area (μm^2^)	Cells area (μm^2^)	nuclear-cytoplasmic ratio	Colony size (mm^2^)
Differentiated cell	155.15 ± 6.60	851.32 ± 11.65	0.182 ± 0.08	52.21 ± 4.73
Control	191.10 ± 5.1*	722.14 ± 16.11*	0.261 ± 0.02*	30.11 ± 5.1*
8*30	195.01 ± 5.2 *	717.11 ± 18.11*	0.263 ± 0.04*	30.28 ± 4.70*
92*50	193.08 ± 6.31 *	721.13 ± 15.08*	0.262 ± 0.07*	31.08 ± 5.3*
92*30	195.02 ± 5.8 *	725.18 ± 17.1*	0.264 ± 0.02*	30.84 ± 3.21*
50*50	192.31 ± 5.65 *	718.14 ± 17.1*	0.263 ± 0.01*	31.15 ± 5.4*
8*50	193.22 ± 5.32 *	7.20 ± 15.12*	0.262 ± 0.03*	30.73 ± 5.13*
50*70	191.8 ± 8.1 *	724.12 ± 16.12*	0.261 ± 0.08*	30.52 ± 6.1*
50*30	195.1 ± 6.61 *	720.16 ± 11.11*	0.264 ± 0.03*	30.55 ± 5.4*
8*70	193.3 ± 5.5 *	722.13 ± 12.1*	0.263 ± 0.04*	30.78 ± 5.32*
92*70	195.2 ± 6.1 *	724.18 ± 15.1 *	0.262 ± 0.04*	31.15 ± 6.13*
111*70	194.2 ± 4.2 *	725.15 ± 13.1*	0.264 ± 0.08*	31.22 ± 4.1*
130*70	157.5 ± 7.3 ^**ns**^	850.15 ± 10.11 ^**ns**^	0.183 ± 0.07 ^**ns**^	51.78 ± 5.21 ^**ns**^

### 3.2 Second step: 24-h cell culture, MTT test, and cell morphology examination

**Fig. 3:**
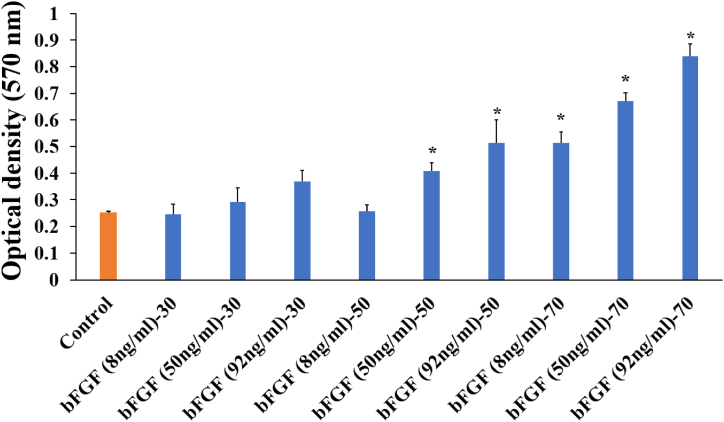
Survival rate (b) (Supplementary Data 2) of the hiPSCs in the nine predicted surface of cultivation conditions after 24 h * indicates significant differences compared to the control group (P < 0.05).

The results of the MTT test and microscopic examination of cultured cells (Fig. 3) showed that the best response was obtained by using a concentration of 92 ng/ml bFGF. Morphological analysis indicated that the cells maintained their normal morphology under a cell density of 70,000 cells/cm^2^ and a bFGF concentration of 92 ng/mL. Healthy stem cells are identifiable by their high nucleus-to-cytoplasm ratio and round or oval-shaped nucleus, setting them apart from differentiated cells (Table 3). They lack apoptotic bodies, which are small vesicles released during programmed cell death (apoptosis). Furthermore, differentiated cell colonies exhibit edge cell morphology and larger size, while stem cell colonies maintain a smaller size and a central core morphology (Table 3). Conversely, at lower cell densities of 50,000 or 30,000 cells/cm^2^ with the same bFGF concentration, differentiation and apoptosis were observed. According to the predicted optimal condition and MTT results, concentrations of 130 ng/ml and 92 ng/ml, as well as the median between the two (111 ng/ml), were chosen for a 48-h culture. Based on the findings of this study, it can be inferred that the addition of bFGF did not significantly alter viability, but the absence of bFGF could potentially compromise pluripotency.

### 3.3 The third stage: 48-h cell culture, expression of pluripotency genes, and examination of cell morphology

**Fig. 4:**
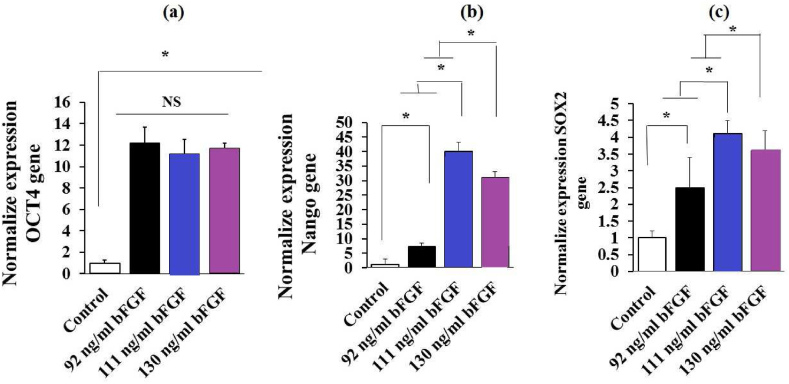
QRT-PCR analysis of pluripotent gene expression levels of OCT4 (a), NANOG (b), and SOX2 (c) in hiPSCs in predicted optimum culture conditions after 48 h of culture. (n = 3 independent samples, data are mean ± SD, and different letters indicate significant differences (P < 0.05). NS: not significant.

After 48 h of culturing hiPSCs in three conditions, the expression of NANOG and SOX2 genes was the highest at a concentration of 111 ng/ml bFGF and a cell density of 70,000 cells/cm2. This was significantly greater than those at the concentrations of 92 or 130 ng/ml bFGF (P < 0.05)(Fig. 4). Cells cultured with 111 ng/ml bFGF exhibited normal morphology, while those cultured with 90 or 130 ng/ml bFGF showed signs of multilayering and differentiation (Table 3). As such, the concentration of 111 ng/ml bFGF was selected for the 7-day culture period.

### 3.4 The fourth stage: final candidate condition of the bFGF concentration and cell density

**Fig. 5:**
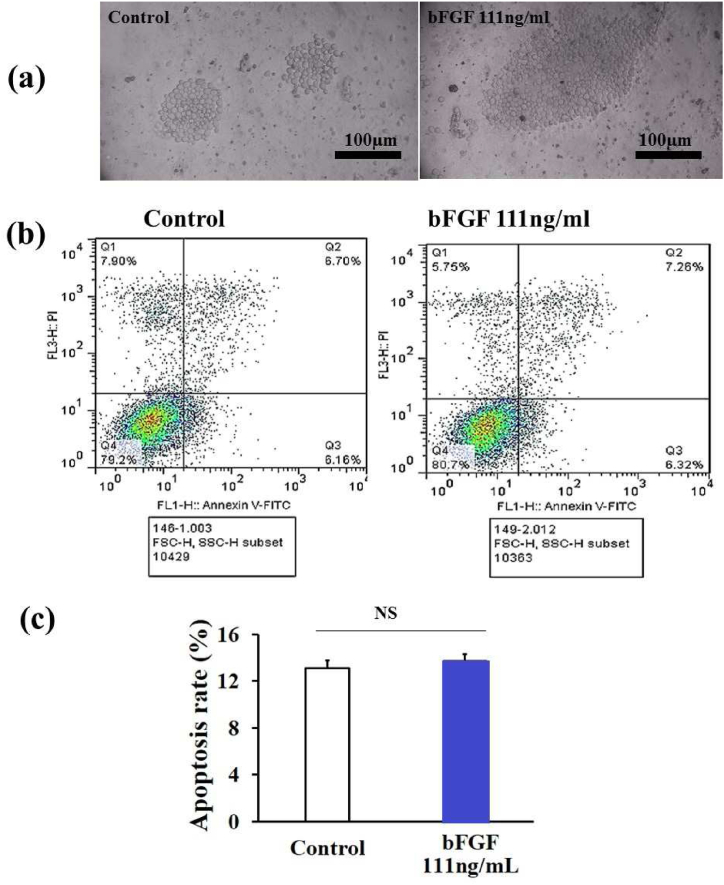
The cell morphology (40 X)(a) and the rate of apoptosis (b and c) induced in the hiPSCs treated with a concentration of 111 ng/ml bFGF at a density of 70,000 cells/cm^2^ were compared to the control group after 7 days of culturing (Supplementary Data 3) (n = 3 independent samples, data are mean ± SD and different letters indicate significant differences (P < 0.05). NS: not significant.

The results of the 7-day culture of the hiPSCs at the concentration of 111 ng/ml bFGF and a density of 70,000 cells/cm^2^ showed that the cells of both treatment and control groups did not differ in terms of cell morphology (Fig. 5, a). Investigating the incidence of apoptosis in the hiPSCs treated with a concentration of 111 ng/ml bFGF and a density of 70,000 cells/cm^2^ did not show any significant difference compared to the control group (P > 0.05)(Table 3)(Fig. 5b and c).

**Fig. 6:**
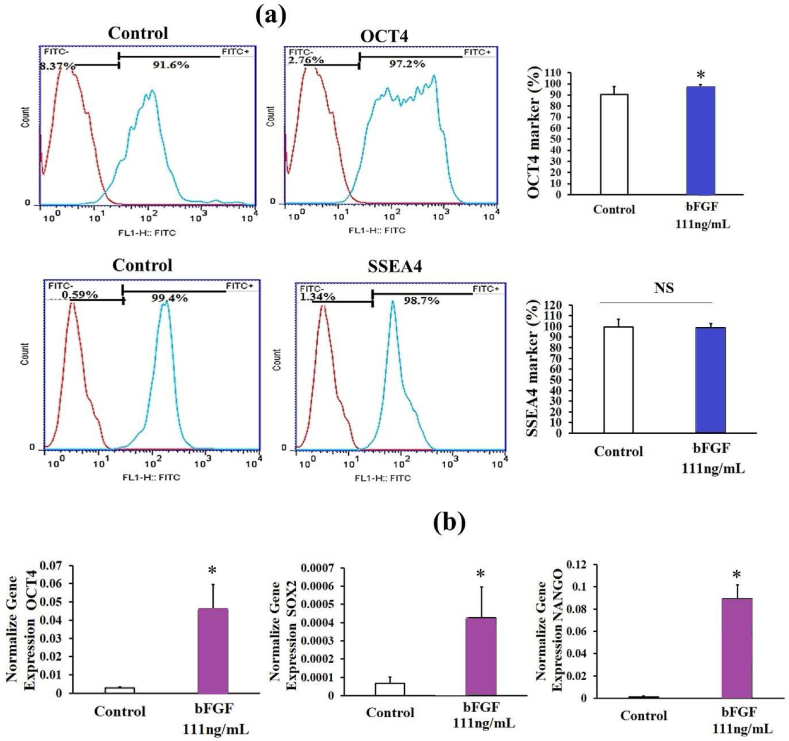
Examination of intracellular (OCT4) and surface (SSEA4) pluripotency markers in hiPSCs in the concentration of 111 ng/ml bFGF and a density of 70,000 cells/cm^2^ after 7 days of culturing (a).The qRT-PCR analysis of pluripotent gene expression levels of OCT4, NANOG, SOX2 in hiPSCs in the concentration of 111 ng/ml bFGF and a density of 70,000 cells/cm2 after 7 days of culturing (b) (Supplementary Data 4). (n = 3 independent samples, data are mean ± SD, and different letters indicate significant differences (P < 0.05). NS: not significant.

Examination of intracellular and surface pluripotency markers revealed that OCT4 in the treatment group was significantly more than the control group (P < 0.05)(Fig. 6a). However, no significant difference was observed in the surface marker of SSEA4 between the control and treatment groups (P > 0.05)(Fig. 6 a). The expression of OCT4, NANOG, and SOX2 genes in the treatment group was significantly higher than the control group (P < 0.05)(Fig. 6 b).

## 4 Discussion

Using DoE approaches for the optimization of stem cell culture can provide a novel differentiation protocol and strategic evidence accumulation [38]. The RSM designs have good potential to reveal novel cell signaling regulation, which is important in both biological aspects and desirable for stable and high-quality cell-differentiated materials [9,38]. In this study, the practical application of RSM design to optimize bFGF concentration and cell density was investigated for the hiPSCs culture.

Several studies have demonstrated that seeding density, a critical process parameter in stem cell culture, can affect significant quality attributes such as specific metabolic rate and specific growth rate [19,39]. This suggests that optimizing the cell density is crucial in preserving essential cell characteristics, minimizing process variability, and maintaining quality attributes [39]. Ratcliffe et al. (2013) utilized a central composite design (CCD) model to optimize the culture condition of human embryonic stem cells (hESCs) by varying the seeding cell density [26].

The bFGF concentration has been known for a long time as a major player in keeping pluripotent stem cells in an undifferentiated state [40]. The addition of bFGF did not significantly alter the viability of hiPSCs in this study, but the absence of bFGF could potentially compromise their pluripotency. A study observed that human placenta-derived cells could support and maintain hPSC characteristics without the exogenous supplementation of growth factors. They developed a conditioned medium from these cells, which could support hPSC growth on a gelatin-coated dish regardless of the presence of bFGF1 [41]. These findings support the inference that the absence of bFGF could compromise the pluripotency of stem cells. Some studies have claimed that the best concentration of the bFGF is 100 ng/mL for maintaining the hiPSCs [42,43]. Previous studies have employed a method of defining the optimal concentration for hiPSC culture that begins with a random selection of concentrations based on prior knowledge, followed by testing each concentration to determine the best readout, typically indicated by the highest proliferation rate [44]. However, this empirical approach has two significant limitations. First, the true optimal concentration may exist in the untested range between the concentrations selected by the experimenters. Second, interactions between factors that may affect the readouts are often overlooked, as these studies optimized one factor at a time. This study addresses these drawbacks by employing a different methodology.

Based on RSM designs, this study claimed that the concentration of 111 ng/mL of the bFGF and the cell density of 70,000 cells/cm^2^ is the optimal concentration for developing the hiPSCs line UMN PCBC16iPS. The introduction of optimal conditions could reduce the number of tests, and the highest proliferation rate of the hiPSCs maintains more potential pluripotency in the hiPSCs. In addition, fine optimization of growth factors known to interfere with pluripotency, e.g. FGF-2, and fine optimization of cell density can limit excessive spontaneous differentiation in the hiPSC culture that interfered with culture maintenance as well as an intended differentiation. This study observed the effects of media change on day 3 after cell passaging, when cells are nearly confluent, and found that optimal systemic media maintained cell pluripotency as effectively as daily feeding. Notably, differences in cell morphology and proliferation were observed between the optimal media and other experimental media, suggesting that empirically formulated media may lack the flexibility to accommodate stressful conditions, thereby limiting the range of tolerance for hiPSCs in this system. Conversely, systematically optimized media can more effectively buffer cellular stress by inducing intrinsic epigenetic modifications in a subset of genes/pathways responsible for promoting flexibility. However, this empirical method also has its drawbacks. The true best concentration or cell density may be in an untested distance between the concentrations and densities chosen by the experimenters. Since only two factors (concentration and cell density) are optimized at a time, the interaction between these factors and other factors that could be effective in readouts is ignored. In hiPSC biomanufacturing, the concentration of bFGF can fluctuate dynamically. The bFGF, being a protein, is often unstable and susceptible to degradation over time due to factors such as temperature [45]. Studies have also indicated that cells can naturally secrete bFGF in smaller amounts than what is added externally [46]. This phenomenon may be challenging to observe in monolayer culture due to the small scale, but it becomes more noticeable in suspension culture with a higher cell density, potentially contributing to cell production on a larger scale for translational applications [47]. The DoE approach has the potential for broader application beyond conventional monolayer culture to include suspension culture. Further consideration of these factors could optimize bFGF usage and improve cost efficiency in production. Therefore, the practical application of RSM design to optimize bFGF concentration and cell density in the hiPSCs culturing requires longer-term validation to show stability over time and throughout longer processes.

Despite significant advancements in optimizing human induced pluripotent stem cell (hiPSC) culture conditions, this study has several limitations. The primary constraint is its focus on only two variables, basic fibroblast growth factor (bFGF) concentration and cell seeding density which may overlook other critical factors affecting hiPSC proliferation and pluripotency. Additionally, the stability and long-term viability of the optimized culture conditions were not thoroughly examined, limiting the applicability of these findings to extended culture processes. Future research should incorporate a broader range of variables and extend the study duration to validate the robustness of the optimized conditions. Furthermore, investigating the interaction of these factors with other culture components could provide a more comprehensive understanding of optimal hiPSC cultivation conditions. Such comprehensive studies would enhance the reproducibility and scalability of hiPSC cultures for clinical and therapeutic applications, thereby advancing the field of regenerative medicine.

## 5 Conclusion

In general, the present study developed a systematically optimized hiPSC culture by application of RSM design that can manipulate and discover the best cell density and the most effective concentration of bFGF that robustly improves culture conditions for the hiPSC. This study presented a novel approach for the efficient and cost-effective optimization of the hiPSCs culturing. During the optimization of hiPSCs culture using this model, the frequency of changing cell media decreased. Furthermore, cells that recover faster after passage experience less stress and are more likely to maintain desirable characteristics over time. Application of RSM design of experiments can generate empirical models that are used predicatively to optimize outputs in a particularly challenging culture system such as the hiPSC culturing. The RSM design can be used as an important tool in creating robust and economical manufacturing processes for cellular therapies.

## Data availability

All data generated or analyzed during this study are included in this published article [and its supplementary information files]

## CRediT authorship contribution statement

**Seyedmilad Mousavi Mirkalaei:** Writing – original draft, Software, Methodology, Investigation, Data curation. **Shirin Farivar:** Writing – original draft, Validation, Supervision, Project administration, Investigation.

## Declaration of competing interest

The authors declare that they have no known competing financial interests or personal relationships that could have appeared to influence the work reported in this paper.
