# Supplementary material for: Systematic optimization of culture media for maintenance of human induced pluripotent stem cells using the response surface methodology
Source: Heliyon. 2024 Jun 9;10(12):e32558. doi: 10.1016/j.heliyon.2024.e32558 (PMC11226774; doi:10.1016/j.heliyon.2024.e32558)
Supplement: Multimedia component 1 [file mmc1.docx]

| **Table S1. Confirmation table of RSM model** |  |  |  |  |  |  |  |  |  |
| --- | --- | --- | --- | --- | --- | --- | --- | --- | --- |

| Response | Predicted Mean | Predicted Median | Observed | Std Dev | n | SE Pred | 95% PI low | Data Mean | 95% PI high |
| --- | --- | --- | --- | --- | --- | --- | --- | --- | --- |
| R1 | 393.556 | 393.556 |  | 16.7111 | 1 | 20.8423 | 327.226 |  | 459.885 |
