# Supplementary material for: Systematic optimization of culture media for maintenance of human induced pluripotent stem cells using the response surface methodology
Source: Heliyon. 2024 Jun 9;10(12):e32558. doi: 10.1016/j.heliyon.2024.e32558 (PMC11226774; doi:10.1016/j.heliyon.2024.e32558)
Supplement: Multimedia component 2 [file mmc2.docx]

**Table S1.** Original data from 24-hour MTT assay

|  | **OD (570 nm)** |  | **Viability rate** | **Viability assay (%)** |  |
| --- | --- | --- | --- | --- | --- |
| **Control** | 0.246 | 0.251 | 0.980079681 | **98.00796813** |  |
| **Control** | 0.257 | 0.251 | 1.023904382 | **102.3904382** |  |
| **Control** | 0.25 | 0.251 | 0.996015936 | **99.60159363** |  |
| **bFGF (8ng/ml)-30** | 0.216 | 0.251 | 0.860557769 | **86.05577689** |  |
| **bFGF (8ng/ml)-30** | 0.287 | 0.251 | 1.143426295 | **114.3426295** |  |
| **bFGF (8ng/ml)-30** | 0.239 | 0.251 | 0.952191235 | **95.21912351** |  |
| **bFGF (50ng/ml)-30** | 0.357 | 0.251 | 1.422310757 | **142.2310757** |  |
| **bFGF (50ng/ml)-30** | 0.279 | 0.251 | 1.111553785 | **111.1553785** |  |
| **bFGF (50ng/ml)-30** | 0.24 | 0.251 | 0.956175299 | **95.61752988** |  |
| **bFGF (92ng/ml)-30** | 0.325 | 0.251 | 1.294820717 | **129.4820717** |  |
| **bFGF (92ng/ml)-30** | 0.377 | 0.251 | 1.501992032 | **150.1992032** |  |
| **bFGF (92ng/ml)-30** | 0.406 | 0.251 | 1.61752988 | **161.752988** |  |
| **bFGF (8ng/ml)-50** | 0.231 | 0.251 | 0.920318725 | **92.03187251** |  |
| **bFGF (8ng/ml)-50** | 0.276 | 0.251 | 1.099601594 | **109.9601594** |  |
| **bFGF (8ng/ml)-50** | 0.267 | 0.251 | 1.06374502 | **106.374502** |  |
| **bFGF (50ng/ml)-50** | 0.379 | 0.251 | 1.509960159 | **150.9960159** |  |
| **bFGF (50ng/ml)-50** | 0.403 | 0.251 | 1.605577689 | **160.5577689** |  |
| **bFGF (50ng/ml)-50** | 0.442 | 0.251 | 1.760956175 | **176.0956175** |  |
| **bFGF (92ng/ml)-50** | 0.602 | 0.251 | 2.398406375 | **239.8406375** |  |
| **bFGF (92ng/ml)-50** | 0.515 | 0.251 | 2.051792829 | **205.1792829** |  |
| **bFGF (92ng/ml)-50** | 0.423 | 0.251 | 1.685258964 | **168.5258964** |  |
| **bFGF (8ng/ml)-70** | 0.48 | 0.251 | 1.912350598 | **191.2350598** |  |
| **bFGF (8ng/ml)-70** | 0.562 | 0.251 | 2.239043825 | **223.9043825** |  |
| **bFGF (8ng/ml)-70** | 0.497 | 0.251 | 1.980079681 | **198.0079681** |  |
| **bFGF (50ng/ml)-70** | 0.706 | 0.251 | 2.812749004 | **281.2749004** |  |
| **bFGF (50ng/ml)-70** | 0.649 | 0.251 | 2.585657371 | **258.5657371** |  |
| **bFGF (50ng/ml)-70** | 0.663 | 0.251 | 2.641434263 | **264.1434263** |  |
| **bFGF (92ng/ml)-70** | 0.867 | 0.251 | 3.454183267 | **345.4183267** |  |
| **bFGF (92ng/ml)-70** | 0.786 | 0.251 | 3.131474104 | **313.1474104** |  |
| **bFGF (92ng/ml)-70** | 0.865 | 0.251 | 3.446215139 | **344.6215139** |  |
|  |  |  |  |  |  |
|  |  |  |  |  |  |
|  |  |  |  |  |  |
|  |  |  |  |  |  |
|  | **Control** | **bFGF (8ng/ml)-30** | **bFGF (50ng/ml)-30** | **bFGF (92ng/ml)-30** | **bFGF (8ng/ml)-50** |
| **Mean** | 100 | 98.54 | 116.3 | 147.1 | 102.8 |
| **Std. Deviation** | 2.218 | 14.43 | 23.73 | 16.35 | 9.487 |
| **Std. Error** | 1.281 | 8.333 | 13.7 | 9.44 | 5.477 |
|  |  |  |  |  |  |
|  | **bFGF (50ng/ml)-50** | **bFGF (92ng/ml)-50** | **bFGF (8ng/ml)-70** | **bFGF (50ng/ml)-70** | **bFGF (92ng/ml)-70** |
| **Mean** | 162.5 | 204.5 | 204.4 | 268 | 334.4 |
| **Std. Deviation** | 12.67 | 35.66 | 17.24 | 11.83 | 18.41 |
| **Std. Error** | 7.314 | 20.59 | 9.955 | 6.833 | 10.63 |

**Table S2. Statistical analysis of the data obtained from the 24-hour MTT test**

| **Table Analyzed** | **Data 1** |  |  |  |  |
| --- | --- | --- | --- | --- | --- |
|  |  |  |  |  |  |
| **One-way analysis of variance** |  |  |  |  |  |
| **P value** | **< 0.0001** |  |  |  |  |
| **P value summary** | ******** |  |  |  |  |
| **Are means signif. different? (P < 0.05)** | **Yes** |  |  |  |  |
| **Number of groups** | **10** |  |  |  |  |
| **F** | **56.68** |  |  |  |  |
| **R square** | **0.9623** |  |  |  |  |
|  |  |  |  |  |  |
| **ANOVA Table** | **SS** | **df** | **MS** |  |  |
| **Treatment (between columns)** | **170505** | **9** | **18945** |  |  |
| **Residual (within columns)** | **6685** | **20** | **334.2** |  |  |
| **Total** | **177189** | **29** |  |  |  |
|  |  |  |  |  |  |
| **Tukey's Multiple Comparison Test** | **Mean Diff.** | **q** | **Significant? P < 0.05?** | **Summary** | **95% CI of diff** |
| **Control vs bFGF (8ng/ml)-30** | **1.461** | **0.1384** | **No** | **ns** | **-51.40 to 54.32** |
| **Control vs bFGF (50ng/ml)-30** | **-16.33** | **1.548** | **No** | **ns** | **-69.19 to 36.53** |
| **Control vs bFGF (92ng/ml)-30** | **-47.14** | **4.467** | **No** | **ns** | **-100.0 to 5.715** |
| **Control vs bFGF (8ng/ml)-50** | **-2.789** | **0.2642** | **No** | **ns** | **-55.65 to 50.07** |
| **Control vs bFGF (50ng/ml)-50** | **-62.55** | **5.926** | **Yes** | ***** | **-115.4 to -9.690** |
| **Control vs bFGF (92ng/ml)-50** | **-104.5** | **9.902** | **Yes** | ******* | **-157.4 to -51.66** |
| **Control vs bFGF (8ng/ml)-70** | **-104.4** | **9.889** | **Yes** | ******* | **-157.2 to -51.52** |
| **Control vs bFGF (50ng/ml)-70** | **-168** | **15.92** | **Yes** | ******* | **-220.9 to -115.1** |
| **Control vs bFGF (92ng/ml)-70** | **-234.4** | **22.21** | **Yes** | ******* | **-287.3 to -181.5** |
| **bFGF (8ng/ml)-30 vs bFGF (50ng/ml)-30** | **-17.8** | **1.686** | **No** | **ns** | **-70.66 to 35.06** |
| **bFGF (8ng/ml)-30 vs bFGF (92ng/ml)-30** | **-48.61** | **4.605** | **No** | **ns** | **-101.5 to 4.254** |
| **bFGF (8ng/ml)-30 vs bFGF (8ng/ml)-50** | **-4.25** | **0.4026** | **No** | **ns** | **-57.11 to 48.61** |
| **bFGF (8ng/ml)-30 vs bFGF (50ng/ml)-50** | **-64.01** | **6.064** | **Yes** | ***** | **-116.9 to -11.15** |
| **bFGF (8ng/ml)-30 vs bFGF (92ng/ml)-50** | **-106** | **10.04** | **Yes** | ******* | **-158.8 to -53.12** |
| **bFGF (8ng/ml)-30 vs bFGF (8ng/ml)-70** | **-105.8** | **10.03** | **Yes** | ******* | **-158.7 to -52.98** |
| **bFGF (8ng/ml)-30 vs bFGF (50ng/ml)-70** | **-169.5** | **16.05** | **Yes** | ******* | **-222.3 to -116.6** |
| **bFGF (8ng/ml)-30 vs bFGF (92ng/ml)-70** | **-235.9** | **22.35** | **Yes** | ******* | **-288.7 to -183.0** |
| **bFGF (50ng/ml)-30 vs bFGF (92ng/ml)-30** | **-30.81** | **2.919** | **No** | **ns** | **-83.67 to 22.05** |
| **bFGF (50ng/ml)-30 vs bFGF (8ng/ml)-50** | **13.55** | **1.283** | **No** | **ns** | **-39.31 to 66.41** |
| **bFGF (50ng/ml)-30 vs bFGF (50ng/ml)-50** | **-46.22** | **4.378** | **No** | **ns** | **-99.07 to 6.645** |
| **bFGF (50ng/ml)-30 vs bFGF (92ng/ml)-50** | **-88.18** | **8.354** | **Yes** | ******* | **-141.0 to -35.32** |
| **bFGF (50ng/ml)-30 vs bFGF (8ng/ml)-70** | **-88.05** | **8.342** | **Yes** | ******* | **-140.9 to -35.19** |
| **bFGF (50ng/ml)-30 vs bFGF (50ng/ml)-70** | **-151.7** | **14.37** | **Yes** | ******* | **-204.5 to -98.80** |
| **bFGF (50ng/ml)-30 vs bFGF (92ng/ml)-70** | **-218.1** | **20.66** | **Yes** | ******* | **-270.9 to -165.2** |
| **bFGF (92ng/ml)-30 vs bFGF (8ng/ml)-50** | **44.36** | **4.202** | **No** | **ns** | **-8.504 to 97.22** |
| **bFGF (92ng/ml)-30 vs bFGF (50ng/ml)-50** | **-15.41** | **1.459** | **No** | **ns** | **-68.26 to 37.45** |
| **bFGF (92ng/ml)-30 vs bFGF (92ng/ml)-50** | **-57.37** | **5.435** | **Yes** | ***** | **-110.2 to -4.511** |
| **bFGF (92ng/ml)-30 vs bFGF (8ng/ml)-70** | **-57.24** | **5.423** | **Yes** | ***** | **-110.1 to -4.378** |
| **bFGF (92ng/ml)-30 vs bFGF (50ng/ml)-70** | **-120.8** | **11.45** | **Yes** | ******* | **-173.7 to -67.99** |
| **bFGF (92ng/ml)-30 vs bFGF (92ng/ml)-70** | **-187.3** | **17.74** | **Yes** | ******* | **-240.1 to -134.4** |
| **bFGF (8ng/ml)-50 vs bFGF (50ng/ml)-50** | **-59.76** | **5.662** | **Yes** | ***** | **-112.6 to -6.901** |
| **bFGF (8ng/ml)-50 vs bFGF (92ng/ml)-50** | **-101.7** | **9.638** | **Yes** | ******* | **-154.6 to -48.87** |
| **bFGF (8ng/ml)-50 vs bFGF (8ng/ml)-70** | **-101.6** | **9.625** | **Yes** | ******* | **-154.5 to -48.73** |
| **bFGF (8ng/ml)-50 vs bFGF (50ng/ml)-70** | **-165.2** | **15.65** | **Yes** | ******* | **-218.1 to -112.3** |
| **bFGF (8ng/ml)-50 vs bFGF (92ng/ml)-70** | **-231.6** | **21.94** | **Yes** | ******* | **-284.5 to -178.7** |
| **bFGF (50ng/ml)-50 vs bFGF (92ng/ml)-50** | **-41.97** | **3.976** | **No** | **ns** | **-94.83 to 10.89** |
| **bFGF (50ng/ml)-50 vs bFGF (8ng/ml)-70** | **-41.83** | **3.963** | **No** | **ns** | **-94.69 to 11.03** |
| **bFGF (50ng/ml)-50 vs bFGF (50ng/ml)-70** | **-105.4** | **9.99** | **Yes** | ******* | **-158.3 to -52.59** |
| **bFGF (50ng/ml)-50 vs bFGF (92ng/ml)-70** | **-171.8** | **16.28** | **Yes** | ******* | **-224.7 to -119.0** |
| **bFGF (92ng/ml)-50 vs bFGF (8ng/ml)-70** | **0.1328** | **0.01258** | **No** | **ns** | **-52.73 to 52.99** |
| **bFGF (92ng/ml)-50 vs bFGF (50ng/ml)-70** | **-63.48** | **6.014** | **Yes** | ***** | **-116.3 to -10.62** |
| **bFGF (92ng/ml)-50 vs bFGF (92ng/ml)-70** | **-129.9** | **12.31** | **Yes** | ******* | **-182.7 to -77.02** |
| **bFGF (8ng/ml)-70 vs bFGF (50ng/ml)-70** | **-63.61** | **6.027** | **Yes** | ***** | **-116.5 to -10.75** |
| **bFGF (8ng/ml)-70 vs bFGF (92ng/ml)-70** | **-130** | **12.32** | **Yes** | ******* | **-182.9 to -77.15** |
| **bFGF (50ng/ml)-70 vs bFGF (92ng/ml)-70** | **-66.4** | **6.291** | **Yes** | ****** | **-119.3 to -13.54** |
