# Supplementary material for: Systematic optimization of culture media for maintenance of human induced pluripotent stem cells using the response surface methodology
Source: Heliyon. 2024 Jun 9;10(12):e32558. doi: 10.1016/j.heliyon.2024.e32558 (PMC11226774; doi:10.1016/j.heliyon.2024.e32558)
Supplement: Multimedia component 3 [file mmc3.docx]

| **Sample** | **Early apoptosis (%)** | **Late apoptosis (%)** | **Total apoptosis (%)** |
| --- | --- | --- | --- |
| **Control** | **6.16** | **6.7** | **12.86** |
| **Control** | **5.48** | **7.1** | **12.58** |
| **Control** | **4.23** | **9.6** | **13.83** |
| **hiPSCs+bFGF (111ng/mL)** | **2.58** | **11.8** | **14.38** |
| **hiPSCs+bFGF (111ng/mL)** | **5.93** | **7.24** | **13.17** |
| **hiPSCs+bFGF (111ng/mL)** | **6.32** | **7.26** | **13.58** |

**Table S1. Original data from Annexin V/PI assay.**

**Table S2. Statistical analysis of early apoptosis.**

| **Table Analyzed** | **Data 1** |
| --- | --- |
| **Column A** | **Control** |
| **vs** | **vs** |
| **Column B** | **hiPS+bFGF (111ng/mL)** |
|  |  |
| **Unpaired t test** |  |
| **P value** | **0.805** |
| **P value summary** | **ns** |
| **Are means signif. different? (P < 0.05)** | **No** |
| **One- or two-tailed P value?** | **Two-tailed** |
| **t, df** | **t=0.2637 df=4** |
|  |  |
| **How big is the difference?** |  |
| **Mean ± SEM of column A** | **5.290 ± 0.5652 N=3** |
| **Mean ± SEM of column B** | **4.943 ± 1.187 N=3** |
| **Difference between means** | **0.3467 ± 1.315** |
| **95% confidence interval** | **-3.303 to 3.996** |
| **R square** | **0.01709** |
|  |  |
| **F test to compare variances** |  |
| **F,DFn, Dfd** | **4.411, 2, 2** |
| **P value** | **0.3696** |
| **P value summary** | **ns** |
| **Are variances significantly different?** | **No** |

**Table S3. Statistical analysis of late apoptosis.**

| **Table Analyzed** | **Data 1** |
| --- | --- |
| **Column A** | **Control** |
| **vs** | **vs** |
| **Column B** | **hiPS+bFGF (111ng/mL)** |
|  |  |
| **Unpaired t test** |  |
| **P value** | **0.6135** |
| **P value summary** | **ns** |
| **Are means signif. different? (P < 0.05)** | **No** |
| **One- or two-tailed P value?** | **Two-tailed** |
| **t, df** | **t=0.5469 df=4** |
|  |  |
| **How big is the difference?** |  |
| **Mean ± SEM of column A** | **7.800 ± 0.9074 N=3** |
| **Mean ± SEM of column B** | **8.767 ± 1.517 N=3** |
| **Difference between means** | **-0.9667 ± 1.767** |
| **95% confidence interval** | **-5.873 to 3.940** |
| **R square** | **0.06958** |
|  |  |
| **F test to compare variances** |  |
| **F,DFn, Dfd** | **2.794, 2, 2** |
| **P value** | **0.5272** |
| **P value summary** | **ns** |
| **Are variances significantly different?** | **No** |

**Table S4. Statistical analysis of total apoptosis.**

| **Table Analyzed** | **Data 1** |
| --- | --- |
| **Column A** | **Control** |
| **vs** | **vs** |
| **Column B** | **hiPS+bFGF (111ng/mL)** |
|  |  |
| **Unpaired t test** |  |
| **P value** | **0.2985** |
| **P value summary** | **ns** |
| **Are means signif. different? (P < 0.05)** | **No** |
| **One- or two-tailed P value?** | **Two-tailed** |
| **t, df** | **t=1.194 df=4** |
|  |  |
| **How big is the difference?** |  |
| **Mean ± SEM of column A** | **13.09 ± 0.3787 N=3** |
| **Mean ± SEM of column B** | **13.71 ± 0.3553 N=3** |
| **Difference between means** | **-0.6200 ± 0.5193** |
| **95% confidence interval** | **-2.062 to 0.8216** |
| **R square** | **0.2627** |
|  |  |
| **F test to compare variances** |  |
| **F,DFn, Dfd** | **1.136, 2, 2** |
| **P value** | **0.9362** |
| **P value summary** | **ns** |
| **Are variances significantly different?** | **No** |
