# Supplementary material for: Systematic optimization of culture media for maintenance of human induced pluripotent stem cells using the response surface methodology
Source: Heliyon. 2024 Jun 9;10(12):e32558. doi: 10.1016/j.heliyon.2024.e32558 (PMC11226774; doi:10.1016/j.heliyon.2024.e32558)
Supplement: Multimedia component 4 [file mmc4.docx]

**Table S1. Original data of OCT4 gene expression measurement in 7-day culture in hiPSCs.**

| Sample | GAPDH | OCT4 | dct | 2^-dct |
| --- | --- | --- | --- | --- |
| Control | 16.4218235 | 24.63472748 | 8.212903976 | 0.003370308 |
| Control | 16.53500748 | 24.9397068 | 8.404699326 | 0.002950757 |
| Control | 15.41199875 | 24.32974815 | 8.917749405 | 0.002067711 |
| hiPSCs+bFGF (111ng/mL) | 16.78193855 | 21.858675 | 5.07673645 | 0.029631255 |
| hiPSCs+bFGF (111ng/mL) | 17.93105698 | 22.13254356 | 4.201486588 | 0.054353374 |
| hiPSCs+bFGF (111ng/mL) | 17.44496727 | 21.68789291 | 4.242925644 | 0.052814371 |

**Table S2. Statistical analysis of OCT4 gene expression measurement in 7-day culture in in hiPSCs**

| Table Analyzed | Data 1 |
| --- | --- |
| Column A | Control |
| vs | vs |
| Column B | bFGF-hipSCs(111ng/mL) |
|  |  |
| Unpaired t test |  |
| P value | 0.0059 |
| P value summary | ** |
| Are means signif. different? (P < 0.05) | Yes |
| One- or two-tailed P value? | Two-tailed |
| t, df | t=5.347 df=4 |
|  |  |
| How big is the difference? |  |
| Mean ± SEM of column A | 0.002796 ± 0.0003839 N=3 |
| Mean ± SEM of column B | 0.04560 ± 0.007997 N=3 |
| Difference between means | -0.04280 ± 0.008006 |
| 95% confidence interval | -0.06503 to -0.02058 |
| R square | 0.8772 |
|  |  |
| F test to compare variances |  |
| F,DFn, Dfd | 433.9, 2, 2 |
| P value | 0.0046 |
| P value summary | ** |
| Are variances significantly different? | Yes |

**Table S3. Original data of NANGO gene expression measurement in 7-day culture in hiPSCs.**

| Sample | GAPDH | NANGO | dct | 2^-dct |
| --- | --- | --- | --- | --- |
| Control | 16.4218235 | 26.22528839 | 9.80346489 | 0.001119085 |
| Control | 16.53500748 | 25.36199188 | 8.826984406 | 0.002201978 |
| Control | 15.41199875 | 25.80732346 | 10.39532471 | 0.000742498 |
| hiPSCs+bFGF (111ng/mL) | 16.78193855 | 20.96984482 | 4.187906265 | 0.054867427 |
| hiPSCs+bFGF (111ng/mL) | 17.93105698 | 20.67301559 | 2.741958618 | 0.149481762 |
| hiPSCs+bFGF (111ng/mL) | 17.44496727 | 21.44198799 | 3.997020721 | 0.062629201 |

**Table S4. Statistical analysis of NANGO gene expression measurement in 7-day culture in in hiPSCs**

| **Table Analyzed** | **Data 1** |
| --- | --- |
| **Column A** | **Control** |
| **vs** | **vs** |
| **Column B** | **bFGF-ipSCs(111ng/mL)** |
|  |  |
| **Unpaired t test** |  |
| **P value** | **0.0446** |
| **P value summary** | ***** |
| **Are means signif. different? (P < 0.05)** | **Yes** |
| **One- or two-tailed P value?** | **Two-tailed** |
| **t, df** | **t=2.889 df=4** |
|  |  |
| **How big is the difference?** |  |
| **Mean ± SEM of column A** | **0.001355 ± 0.0004375 N=3** |
| **Mean ± SEM of column B** | **0.08899 ± 0.03033 N=3** |
| **Difference between means** | **-0.08764 ± 0.03033** |
| **95% confidence interval** | **-0.1718 to -0.003441** |
| **R square** | **0.6761** |
|  |  |
| **F test to compare variances** |  |
| **F,DFn, Dfd** | **4806, 2, 2** |
| **P value** | **0.0004** |
| **P value summary** | ******* |
| **Are variances significantly different?** | **Yes** |

**Table S5. Original data of SOX2 gene expression measurement in 7-day culture in hiPSCs.**

| Sample | GAPDH | SOX2 | dct | 2^-dct |
| --- | --- | --- | --- | --- |
| Control | 16.4218235 | 29.99923325 | 13.57740974 | 8.18074E-05 |
| Control | 16.53500748 | 29.94556618 | 13.4105587 | 9.18374E-05 |
| Control | 15.41199875 | 30.48344994 | 15.07145119 | 2.9043E-05 |
| hiPSCs+bFGF (111ng/mL) | 16.78193855 | 28.66017723 | 11.87823868 | 0.00026564 |
| hiPSCs+bFGF (111ng/mL) | 17.93105698 | 29.17650223 | 11.24544525 | 0.000411892 |
| hiPSCs+bFGF (111ng/mL) | 17.44496727 | 28.14133072 | 10.69636345 | 0.000602662 |

**Table S6. Statistical analysis of SOX2 gene expression measurement in 7-day culture in in hiPSCs**

| **Table Analyzed** | **Data 1** |
| --- | --- |
| **Column A** | **Control** |
| **vs** | **vs** |
| **Column B** | **bFGF-ipSCs(111ng/mL)** |
|  |  |
| **Unpaired t test** |  |
| **P value** | **0.0226** |
| **P value summary** | ***** |
| **Are means signif. different? (P < 0.05)** | **Yes** |
| **One- or two-tailed P value?** | **Two-tailed** |
| **t, df** | **t=3.610 df=4** |
|  |  |
| **How big is the difference?** |  |
| **Mean ± SEM of column A** | **6.756e-005 ± 1.948e-005 N=3** |
| **Mean ± SEM of column B** | **0.0004267 ± 9.757e-005 N=3** |
| **Difference between means** | **-0.0003592 ± 9.950e-005** |
| **95% confidence interval** | **-0.0006354 to -8.296e-005** |
| **R square** | **0.7651** |
|  |  |
| **F test to compare variances** |  |
| **F,DFn, Dfd** | **25.10, 2, 2** |
| **P value** | **0.0766** |
| **P value summary** | **ns** |
| **Are variances significantly different?** | **No** |
